# ^18^F-DCFPyL PSMA PET/CT Tracheobronchial Uptake in Patients with Prostate Cancer: Incidence and Etiology

**DOI:** 10.2967/jnumed.124.267772

**Published:** 2024-09

**Authors:** Medhat M. Osman, Amir Iravani, Catherine Mitchell, Rodney J. Hicks, Elisa Perry, Michael S. Hofman

**Affiliations:** 1 Department of Radiology, Saint Louis University Hospital, St. Louis, Missouri;; 2 Department of Radiology, Fred Hutchinson Cancer Center, University of Washington, Seattle, Washington;; 3 Department of Pathology, Peter MacCallum Cancer Centre, Melbourne, Victoria, Australia;; 4 Melbourne Theranostic Innovation Centre, Melbourne, Victoria, Australia;; 5 Department of Cancer Imaging, Peter MacCallum Cancer Centre, Melbourne, Victoria, Australia; and; 6 Sir Peter MacCallum Department of Oncology, University of Melbourne, Melbourne, Victoria, Australia

**Keywords:** trachea, prostate cancer, ^18^F-DCFPyL, PET/CT, submucosal glands

## Abstract

We evaluated the incidence and potential etiology of tracheobronchial uptake in patients being evaluated by ^18^F-DCFPyL PET/CT for prostate cancer (PCa). **Methods:** The study included a consecutive 100 PCa patients referred for ^18^F-DCFPyL PET/CT. The PET/CT scans were retrospectively reviewed. The presence or absence of physiologic tracheobronchial uptake on PET/CT was recorded. To further evaluate tracheal prostate-specific membrane antigen (PSMA) expression, immunohistochemistry was performed on tracheal samples taken from 2 men who had surgical resection of lung cancer. **Results:** Tracheal uptake was present in 31 of 100 patients (31%). When tracheal uptake was present, the SUV_max_ was significantly higher in the left main bronchus (mean, 2.7) than in the right (mean, 2.3) (*P* < 0.001). Histopathologic testing of tracheobronchial samples showed PSMA expression in bronchial submucosal glands. **Conclusion:** In PCa patients undergoing ^18^F-DCFPyL PET/CT, tracheobronchial uptake occurred in 31% of patients. This is attributed to normal physiologic PSMA expression in bronchial submucosal glands.

## Visual Abstract

**Figure:**
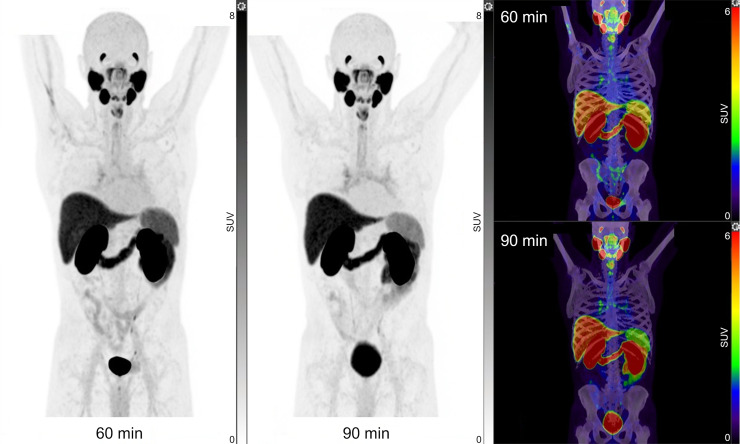

Despite the promise of prostate-specific membrane antigen (PSMA) uptake, it is not completely specific to prostate cancer (PCa), with reports of uptake in benign and non-PCa malignancies as well (1–3). At our center, we have noted that in the trachea and bronchi, ^18^F-DCFPyL PET/CT uptake appears to be more common than ^68^Ga PSMA-11 uptake, but this finding has been described only as case reports in the literature to date (4–8). In this work, we aimed to determine the frequency with which tracheobronchial uptake is encountered in PCa patients undergoing ^18^F-DCFPyL PET/CT and its relationship to uptake in other normal organs, as well as to provide a histopathologic explanation for such uptake.

## MATERIALS AND METHODS

### Patient Population

The study included 100 consecutive PCa patients referred for ^18^F-DCFPyL PET/CT. This retrospective study was approved by the Institutional Human Research Ethics Committee with a waiver of informed consent from patients who had been scanned for clinical indications.

### ^18^F-DCFPyL PET/CT Image Acquisition and Protocol

The ^18^F-DCFPyL PET/CT image acquisition and protocol are described in the supplemental materials (available at http://jnm.snmjournals.org).

### Data Analysis

For each of the 100 PCa patients who had ^18^F-DCFPyL scans, we collected data on age, prostate-specific antigen (PSA) level, injected activity, and length of uptake phase. For each scan, imaging data were analyzed using MIM Encore (MIM Software Inc.). Maximum-intensity projection images were visually assessed for the presence or absence of PSMA uptake above the blood pool level in the trachea and main bronchi and semiquantitatively by SUV_max_. Also, SUV_max_ measurements were obtained for normal physiologic uptake in the lacrimal gland, parotid gland, submandibular gland, liver, spleen, kidney, small bowel, left and right stellate and celiac ganglia, blood pool, and gluteal region. Normal-organ uptake was compared in patients with and without tracheal uptake. The collected data for measured parameters were analyzed for statistical significance using a paired *t* test. To illustrate and compare tracheal ^68^Ga-PSMA-11 and ^18^F-DCFPyL uptake in the same patient, PET/CT images using both tracers were evaluated in only one patient.

### Immunohistochemistry

To confirm tracheobronchial PSMA expression, immunohistochemistry was performed on tracheobronchial samples taken from 2 men who had surgical resection of lung cancer; the supplemental materials provide immunohistochemistry details.

## RESULTS

**FIGURE 1.**
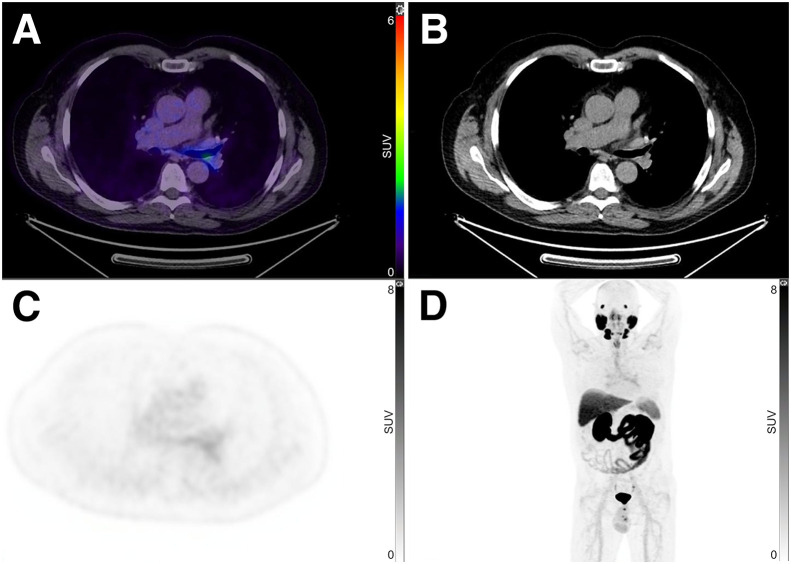
^18^F-DCFPyL fused axial PET/CT (A), CT (B), PET (C), and PET maximum-intensity projection (D) showing tracheal uptake.

**FIGURE 2.**
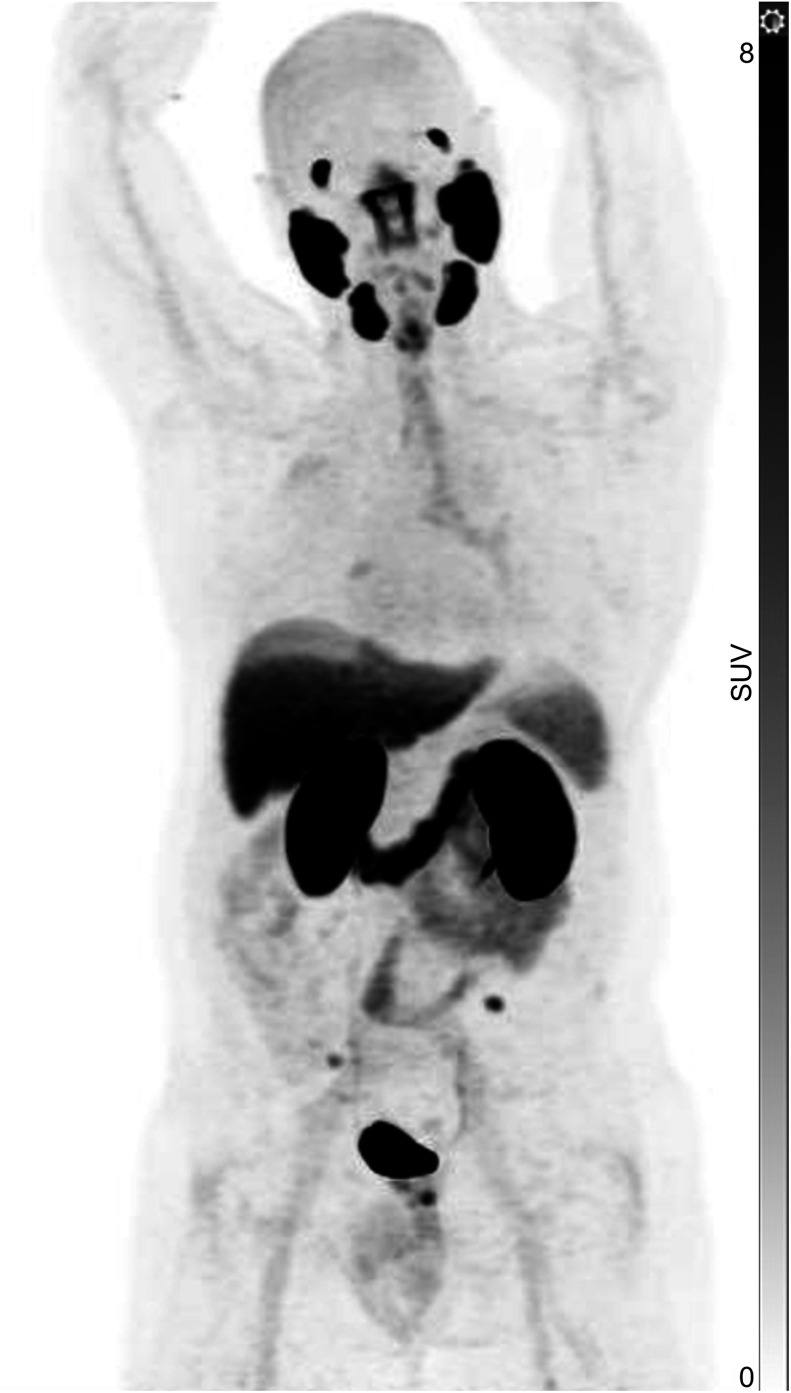
^18^F-DCFPyL PET maximum-intensity projection showing tracheal uptake to be significantly higher in left main bronchus.

**FIGURE 3.**
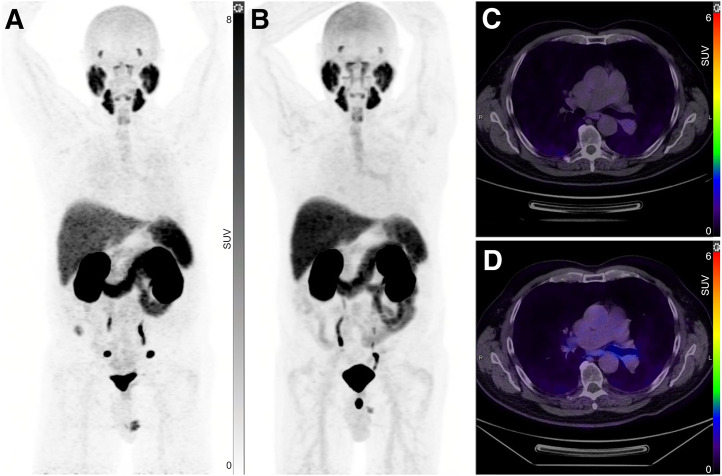
Baseline ^68^Ga-PSMA-11 and 1 y posttherapy ^18^F-DCFPyL images for same patient. (A and C) ^68^Ga-PSMA-11 PET maximum-intensity projection (A) and fused axial PET/CT image (C) showing minimal tracheal uptake 60 min after injection. (B and D) ^18^F-DCFPyL PET maximum-intensity projection (B) and fused axial PET/CT image (D) showing relatively higher tracheal uptake 90 min after injection.

**FIGURE 4.**
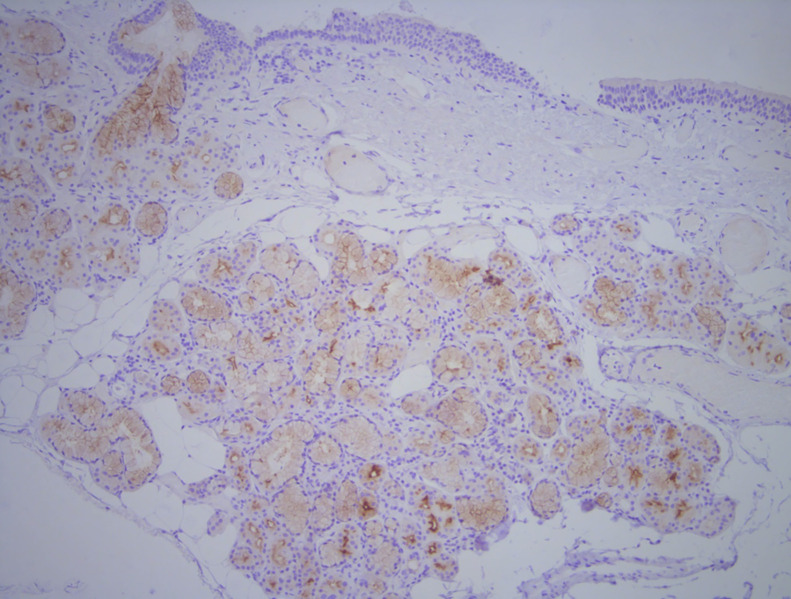
Immunohistochemistry for PSMA showing weak to moderate reactivity within bronchial glands (seen as brown staining, predominantly cytoplasmic distribution with luminal accentuation in ducts) (×100).

Of the 100 patients (mean age, 67.4 y [SD, 6.68 y]; range, 50–85 y), tracheobronchial uptake was present in 31 (31%) (Fig. 1). Paired *t* testing showed that when tracheal uptake was present, the SUV_max_ was significantly higher in the left main bronchus (mean, 2.7) than in the right (mean, 2.3) (*P* < 0.001) (Fig. 2). Also, patients with tracheobronchial uptake showed significantly higher PSMA uptake in the lacrimal and submandibular glands but lower PSA and uptake in the blood pool (Table 1). However, there were no significant differences in age, injected PSMA activity, duration of uptake phase, or other measured areas of uptake, including the parotid gland, liver, spleen, kidney, small bowel, ganglia (stellate or celiac), and gluteal muscle. ^68^Ga-PSMA-11 and ^18^F-DCFPyL in the same patient revealed more prominent tracheal uptake of ^18^F-DCFPyL (Fig. 3). Immunohistochemistry performed on tracheobronchial samples taken from 2 lung cancer patients demonstrated weak-to-moderate PSMA expression in bronchial submucosal glands in both cases (Fig. 4).

**TABLE 1.** Tracheal PSMA Uptake Groups

Parameter	Tracheal uptake (left or both sides) (*n* = 31)	No tracheal uptake (*n* = 69)	*P*
Age (y)	67.39 (5.86)	67.42 (7.06)	0.98
^18^F-DCFPyL dose (MBq)	325.00 (41.09)	311.96 (54.49)	0.24
Uptake (min)	102.58 (25.740	94.13 (20.19)	0.11
PSA	6.63 (9.46)	21.24 (48.19)	0.02*
Lacrimal	12.96 (4.72)	10.95 (4.49)	0.04*
Parotid	18.78 (5.84)	17.55 (6.51)	0.37
Submandibular	21.30 (6.78)	17.27 (6.56)	0.006*
Liver	8.24 (1.33)	8.29 (2.24)	0.86
Spleen	6.57 (3.20)	6.43 (2.78)	0.84
Renal	46.98 (13.53)	43.63 (15.03)	0.29
Small bowel	16.32 (4.87)	14.13 (7.91)	0.16
L stellate	1.00 (1.18)	0.89 (1.07)	0.63
R stellate	0.47 (0.82)	0.60 (0.94)	0.51
L celiac	0.77 (1.07)	0.51 (1.01)	0.25
R celiac	0.14 (0.56)	0.30 (0.80)	0.33
Blood pool	1.65 (0.25)	1.82 (0.43)	0.04*
Gluteal	0.60 (0.14)	0.61 (0.17)	0.75

* Significant at *P* < 0.05.

Values are reported as mean followed by SD in parentheses for each variable within tracheal uptake group. *P* value is based on independent-samples *t* test.

## DISCUSSION

PSMA PET/CT is now considered the new standard of care in the management of PCa, with inclusion of PSMA PET/CT in the National Comprehensive Cancer Network guidelines (9). Despite the high diagnostic accuracy of PSMA-based agents in various PCa scenarios, PSMA is not specific to PCa or even to the prostatic gland. Several studies have shown a wide spectrum of nonprostatic benign and malignant PSMA uptake on PET using virtually all PSMA-based agents. We noted an unusual pattern in which physiologic uptake on PSMA PET was frequently seen in the trachea and proximal bronchi, on the left more than the right. Published examples of this pattern of PSMA tracheal uptake are limited to case reports, primarily attributed to underlying lung pathology, and do not localize the site of or determine the mechanism of PSMA expression (4–8).

In the present study, tracheal uptake was noted in 31% of patients undergoing ^18^F-DCFPyL PET/CT (Fig. 1). No relationship was found between tracheobronchial uptake and age, injected activity of ^18^F-DCFPyL, or uptake in the parotid gland, liver, spleen, kidney, small bowel, stellate or celiac ganglia, or gluteal muscle (Table 1). However, those with tracheobronchial uptake had significantly higher uptake in the lacrimal and submandibular glands but lower uptake in the blood pool and a lower PSA level. The higher uptake in the salivary glands and trachea might be related to their being secretory glands with a similar origin. Although xerostomia is one of the most common side effects of PSMA radioligand therapy, we could find no report that PSMA therapy can cause adverse airway events or respiratory symptoms. In our study, a lower PSA level was associated with higher tracheobronchial uptake. This might reflect a lower sink effect, with a low PSA level generally indicating a lower disease burden. However, lower blood pool uptake may contribute to a higher physiologic-to-background ratio, thus resulting in more noticeable tracheal uptake. Higher tracheobronchial uptake was also associated with lower uptake in the blood pool. This may reflect uptake time, as blood pool clearance and accumulation of tracer in PSMA-expressing tissues increase with time. The trachea receives its nerve supply from the pulmonary plexus, which is derived from both the sympathetic and the parasympathetic nervous systems. We hypothesized that in patients with higher autonomic tone, higher tracheal uptake may be noted more frequently in those with higher ganglia uptake, particularly stellate, since it has the same nerve supply as the trachea. In our study, tracheal uptake was associated with higher uptake in the left stellate and left celiac ganglia than in the right stellate and right celiac ganglia. Also, tracheal uptake was associated with higher lacrimal and submandibular uptake. Such a pattern might be suggestive of an underlying higher autonomic tone in the trachea, particularly on the left side. Paired *t* testing showed that when tracheal uptake was present, the SUV_max_ was significantly higher in the left main bronchus (mean, 2.7) than in the right (mean, 2.3) (*P* < 0.001). Anatomic differences between the narrower, longer, and more horizontal left bronchus and the wider, shorter, and more vertical right bronchus might also contribute to more PSMA PET visualization on the left side than on the right (Fig. 2).

Several studies showed differences in biodistribution between currently Food and Drug Administration–approved PSMA agents (10–12). Our data show that a relatively higher injected activity and longer uptake duration may lead to higher contrast in the image as well. In our experience, tracheobronchial uptake is seen relatively more often on ^18^F-DCFPyL PET/CT than on ^68^Ga-PSMA-11 PET/CT (Fig. 3), but this may be because of higher contrast related to the higher positron yield and lower positron energy of ^18^F or because we tend to image later (90 min after injection) and inject higher activity with ^18^F-PSMA agents. More recently, we changed our ^18^F-DCFPyL imaging protocol from 90 to 60 min, which may lead to less detection of the tracheobronchial uptake. We made this change to align with Food and Drug Administration prescribing information.

Recent studies reported PSMA expression in small salivary glands in the nasopharynx (13). To localize tracheobronchial PSMA expression, immunohistochemistry was performed on tracheobronchial samples taken from 2 men who had surgical resection of lung cancer. Figure 4 shows PSMA expression in bronchial submucosal glands, suggesting that this uptake is related to tracheoglandular function. Submucosal glands are a prominent structure that lines human cartilaginous airways and contributes to respiratory defense in protecting the lung (14*,*^15^). This explains why tracheobronchial PSMA uptake is frequently seen in the absence of morphologic or metabolic changes suggestive of an inflammatory condition but more prominent uptake is noticed in the presence of underling conditions that may cause more reactive changes at the submucosal gland. In chronic obstructive pulmonary disease and asthma, hypertrophied bronchial submucosal glands are well documented (16). Such hypertrophy may lead to more prominent tracheal PSMA uptake. In general, PSMA tracheobronchial uptake is mild and relatively diffuse. It is rarely focal and is therefore not likely to represent a source of false-positive interpretation. Similar to other well-documented physiologic uptake, however, the clinical significance of tracheobronchial uptake is not yet known.

The present study was limited by the retrospective analysis. We did not assess the relationship of tracheal uptake to asthma, smoking, or occupational exposure. Furthermore, we did not evaluate the impact of switching from a 90- to 60-min uptake phase on the frequency with which tracheobronchial ^18^F-DCFPyL PET/CT uptake is detected, nor did we systematically compare detection with other PSMA agents.

## CONCLUSION

Tracheobronchial uptake occurs in approximately one third of PCa patients undergoing ^18^F-DCFPyL PET/CT. The histopathologic correlation with PSMA expression in bronchial submucosal glands appears to explain the etiology of PET findings but may be driven by increased autonomic tone.

## DISCLOSURE

Michael Hofman acknowledges philanthropic/government grant support from the Prostate Cancer Foundation including CANICA Oslo Norway, the Peter MacCallum Foundation, and the Medical Research Future Fund (NHMRC investigator grant). Unrelated to this article, Michael Hofman acknowledges research grant support (to the Peter MacCallum Cancer Centre) from Novartis (including AAA and Endocyte), ANSTO, Bayer, Isotopia, and MIM and consulting fees for lectures or advisory boards from Astellas, AstraZeneca, and MSD in the last 2 y. Rodney Hicks is a shareholder in Telix Pharmaceuticals; a consultant to GE Healthcare; on the speakers’ board of Siemens Healthineers; and the founder, executive director, and a major shareholder of PreMIT Pty Ltd. and Precision Molecular Imaging and Theranostics Pty Ltd. Medhat Osman acknowledges research grant support (to Saint Louis University and Saint Louis VA) from Novartis, POINT Biopharma, and Curium Pharma and is on the speakers’ bureau of Lantheus. No other potential conflict of interest relevant to this article was reported.

## Notes

KEY POINTS **QUESTION:** What is incidence and potential etiology of tracheobronchial uptake in patients being evaluated by ^18^F-DCFPyL PET/CT? **PERTINENT FINDINGS:** Tracheobronchial uptake occurs in approximately one third of PC patients undergoing ^18^F-DCFPyL PET/CT. The histopathologic correlation with PSMA expression in bronchial submucosal glands appears to explain the etiology of PET findings but may be driven by increased autonomic tone. **IMPLICATIONS FOR PATIENT CARE:** In general, PSMA tracheobronchial uptake is mild and relatively diffuse. It is rarely focal and therefore is not likely to represent a source of false-positive interpretation.
